# Strategies for Assessing Physical Compatibility of Calcium Folinate with Bicarbonate During Methotrexate Rescue Therapy in Pediatric Patients with Acute Lymphoblastic Leukemia

**DOI:** 10.3390/pharmaceutics17091155

**Published:** 2025-09-03

**Authors:** Kaveh Teimori, Bjarke Strøm Larsen, Mathias Buaas Austli, Niklas Nilsson, Ingunn Tho, Katerina Nezvalova-Henriksen

**Affiliations:** 1 Oslo Hospital Pharmacy, Hospital Pharmacies Enterprise, South-Eastern Norway, 0372 Oslo, Norway; mathias.buaas.austli@sahf.no (M.B.A.); niklas.nilsson@sahf.no (N.N.); katerina.nezvalova-henriksen@sahf.no (K.N.-H.); 2 Department of Pharmacy, University of Oslo, 0316 Oslo, Norway; b.s.larsen@farmasi.uio.no; 3 Department of Hematology, Oslo University Hospital, South-Eastern Norway, 0372 Oslo, Norway

**Keywords:** rescue therapy, clinical pharmaceutics, co-administration, calcium folinate, bicarbonate, acute lymphoblastic leukemia, pediatric, Raman spectroscopy, precipitate identification, analytical quality control

## Abstract

**Background/Objectives**: Acute lymphoblastic leukemia (ALL) is the most prevalent childhood cancer requiring cytotoxic methotrexate treatment. This always necessitates intravenous administration of rescue therapy consisting of calcium folinate and bicarbonate. Current recommendations advise against mixing these two drugs due to concerns regarding precipitate formation of calcium carbonate (CaCO_3_) that could result in catheter and capillary obstruction. These recommendations are based on drug concentrations not clinically relevant in pediatric ALL settings. Our study investigated the effect of clinically relevant calcium folinate–bicarbonate concentrations on the risk of CaCO_3_ precipitation. **Methods**: A theoretical prediction model provided estimates of final mixing concentrations in five scenarios: three simulated pediatric patient models (approx. 1, 9, and 14 years), an undiluted drug mix, and a high-risk control outlier case. Physical compatibility tests were conducted using validated methods for particle detection, complemented by Raman spectroscopy for particle identification. **Results**: Theoretical predictions suggested CaCO_3_ precipitation with elevated bicarbonate concentrations and pH levels. Our simulated patient models and high-risk control outlier case showed that CaCO_3_ precipitation may be avoided below certain serum methotrexate concentrations and thereby calcium folinate and bicarbonate concentrations. Physical testing demonstrated particle formation only in the undiluted mix with Raman spectroscopy confirming the finding. **Conclusions**: Mixing calcium folinate and bicarbonate appears safe under specific methotrexate-directed pediatric ALL treatment conditions. While high bicarbonate concentrations pose precipitation risks, protocol-based dosing regimens mitigate this. Switching to disodium folinate or using in-line filters could further enhance co-administration safety if bicarbonate concentrations exceed the safety limit suggested by our results.

## 1. Introduction

The most commonly occurring childhood cancers are acute leukemias [1]. Acute lymphoblastic leukemia (ALL) accounts for almost a quarter of these diagnoses, especially in children between one and four years of age [1,2]. One of the many cytotoxic drugs used to treat ALL is the folate antimetabolite methotrexate (MTX). Supportive drugs are administered simultaneously, most intravenously (IV) [3,4,5,6,7,8,9,10,11,12,13,14,15]. The treatment regimen starts with MTX and on the second day of the treatment regimen, the widely utilized pediatric “ALL-together” protocol [15] prescribes rescue therapy, which aims at counteracting MTX toxic effects and completely clearing the cytostatic agent from the patient’s bloodstream [16]. Calcium folinate (or leucovorin calcium) [17], an MTX neutralizing agent, and a rehydration fluid with added sodium bicarbonate (NaHCO_3_), to increase MTX renal excretion rate by increasing the pH of urine, are standard of care for this purpose [18]. To ensure optimal calcium folinate dosing, the patient’s serum MTX concentrations (s-MTX) are regularly monitored [15,19,20].

The two solutions are administered through a centrally inserted venous catheter (CVC) [21,22] or a peripherally inserted central venous catheter (PICC) [23]. In pediatric patients, these IV catheters may consist of one or two parallel lumen (internal channels) [24], meaning there are two separate points of entry into the systemic bloodstream. Since the number of prescribed drugs typically outnumbers the available lumens, drug solutions are commonly co-infused through the same lumen and thereby mixed. In the case of ALL therapy, co-administration of calcium folinate and NaHCO_3_ would mean mixing calcium folinate (Ca^2+^) with varying proportions of bicarbonate (HCO_3_)^−^ and its conjugate base carbonate (CO_3_^2−^). It is well known that mixing Ca^2+^ with species of carbonate can lead to the formation of the precipitate CaCO_3_ [25,26,27,28,29]. Since HCO_3_^−^ and CO_3_^2−^ are part of the carbonic acid (H_2_CO_3_) buffer system, their concentrations both influence and depend on the pH of the resultant solution. Hence, the final pH of the mixed solutions, together with the concentrations/dose of both calcium folinate and sodium bicarbonate, have the potential to determine whether the poorly soluble calcium carbonate (CaCO_3_) salt will form upon mixing. If the concentrations of Ca^2+^ and CO_3_^2−^ in solution exceed the solubility limit CaCO_3_ (0.0166 mg/mL [30]), precipitation will occur. This may happen inside the lumen of the infusion set and could lead to either occlusion of the catheter line [31,32] or, if the particles enter the patient’s bloodstream, occlusion of the capillaries. Infusion of solid particles has been associated with fatal outcomes [33,34,35], and precipitation of solid Ca^2+^ drug particles due to mixing of intravenous fluids has led to the formation of emboli in both systemic and pulmonary capillaries in pediatric patients [36,37,38,39]. In fact, the mixture of calcium folinate and HCO_3_^−^ has been reported to cause precipitation of CaCO_3_ within the infusion set in adults [40,41,42,43]. Compatibility databases therefore advise against mixing these two drugs [17,44,45], and these recommendations are adhered to by hospital pharmacists, nurses, and doctors managing the MTX rescue therapy. One often cited study, ref. [46] reported precipitation after mixing undiluted calcium folinate (10 mg/mL) and sodium bicarbonate (167 mmol/L) at a 1:1 volume mixing ratio. This corresponds to HCO_3_^−^ concentrations that exceed pediatric protocol specifications [15]. Since protocol-based dosing requires administration of fluids at different rates and drug concentrations, the applicability of this finding to specific clinical cases can be impractical and limiting [47]. Additionally, the authors do not explicitly specify the nature of the precipitate [46]. This means that their conclusion leaves room for speculation as to which substance or excipient in their mixture is the culprit and which corresponding concentrations and infusion rates should be avoided. To fully predict compatibility, studies need to be performed under clinically relevant infusion conditions taking into account dilution and infusion rates, as well as changes in pH [47,48,49,50]. Due to the fear of co-administering calcium folinate and bicarbonate intravenously, pediatric wards struggle with sufficient intravenous entry points, as these two fluids are given separately. If calcium folinate and bicarbonate could be safely co-administered in the same lumen this problem would be solved.

This study investigated whether, and if so, how different y-site concentrations of Ca^2+^, HCO_3_^−^, and CO_3_^2−^ dictated by the ALL-together protocol, lead to the precipitation risk of CaCO_3_. Firstly, a theoretical evaluation strategy was applied to estimate relevant mixing ratios and concentrations of key components in five simulated models: three simulated patient models (age approx. 1, 9, and 14 years), one undiluted mix of calcium folinate (10 mg/mL) and sodium bicarbonate (500 mmol/L, and one hypothetical high-risk control outlier case, designated “extreme patient”. Secondly, the identified mixing ratios were challenged by exploring what was happening to the estimated y-site concentrations and risk of CaCO_3_ precipitation in a dynamically changing environment directed by the ALL-together protocol. Finally, in vitro compatibility testing was conducted by simulating the predicted mixing ratios of the patient models and the undiluted mix, using a battery of validated methods [51] in addition to Raman spectroscopy to explore its aptness for identifying the nature of any particles discovered.

## 2. Materials and Methods

### 2.1. Test Materials

Calcium folinate 10 mg/mL, pH 6.5–8.5 [17,19,44] (Pfizer, Zaventem, Belgium), Sodium hydrogen carbonate 500 mmol/L (sodium bicarbonate), pH 7.0–8.5 [52] (B. Braun, Melsungen, Germany), sodium chloride 9 mg/mL, pH 4.5–7.0 [53] (B. Braun, Melsungen, Germany), and standard glucose 50 mg/mL solution containing sodium chloride 2.3 mg/mL (Na^+^ 40 mmol/L; Cl^−^ 40 mmol/L) and potassium chloride 1.5 mg/mL (K^+^ 20 mmol/L; Cl^−^ 20 mmol/L), pH 4 [54] (B. Braun, Melsungen, Germany) were procured from the pediatric cancer clinic at Oslo University Hospital. Anhydrous calcium carbonate (CaCO_3_) standard (Merck, Darmstadt, Germany) was used as reference material in the Raman analysis.

Ultra purified water (Merck Millipore, Merck, Darmstadt, Germany), termed Milli-Q^®^ (MQ) water, was used for preparation of samples and as a particle-free reference in in vitro analyses. The MQ system included a Millipak^®^ Express filter component containing a flat polyethersulfone (PES) 0.2 µm pore size membrane, for the removal of particles and microorganisms from the purified water.

### 2.2. Modelling Y-Site Concentrations in Rescue Therapy

**Figure 1:**
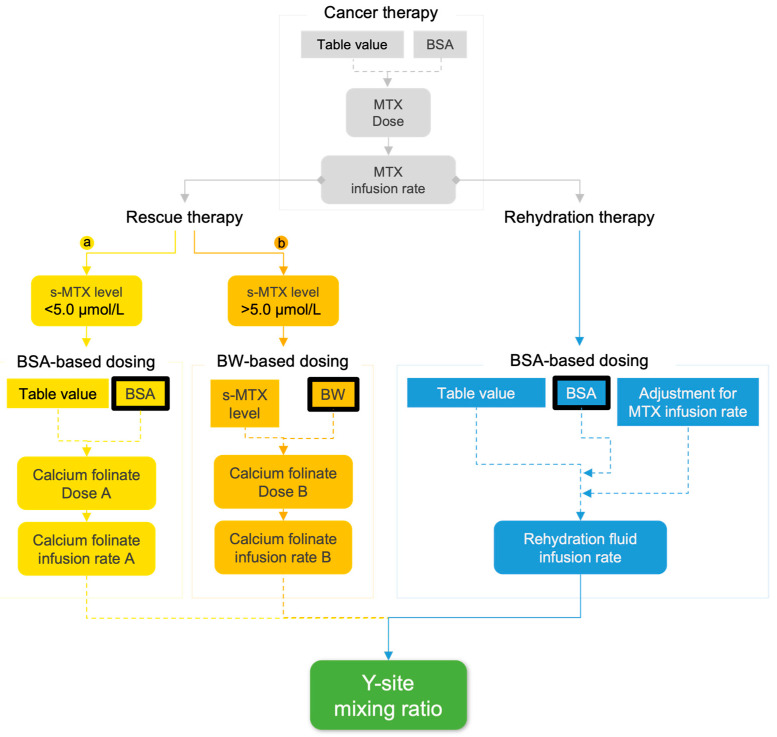
The complex and dynamic relationship between dosing, infusion rates, concentrations, and how they influence mixing ratios of the rescue therapy (calcium folinate) and the HCO_3_^−^-containing rehydration fluid, inside the catheter lumen (internal channel) at y-site, based on the pediatric ALL-together protocol [15]. The calcium folinate dosing was based on either body surface area (BSA) in cases where serum methotrexate concentrations (s-MTX) were <5.0 µmol/L (a), or bodyweight (BW), for cases where s-MTX was >5.0 µmol/L (b). The rehydration therapy was BSA-based and was adjusted according to the MTX infusion rate to achieve a total infusion rate of 3000 mL/m^2^/day (MTX + rehydration fluid). Table values refer to standardized dosing and administration guidelines prescribed by the protocol [15].

The complex and dynamic dosing schedule [10] during induction of rescue therapy involved in the studied pediatric ALL-together protocol [15] is related to patient bodyweight (BW), body surface area (BSA), methotrexate (MTX) dose, MTX infusion rate, and s-MTX levels. Since these parameters all impact infusion rates throughout the entire treatment, the resulting y-site infusion rate ratios (i.e., mixing ratios) for calcium folinate and bicarbonate (HCO_3_^−^) were estimated with a model (Figure 1) to allow for predictions of the final y-site concentrations in different patient models or cases. Initially, the MTX infusion rate was predicted based on a 24 h administration time and at a dose of 500 mg/m^2^ for the first hour (50 mL), followed by 4500 mg/m^2^ for 23 h (500 mL). The infusion rate of the rehydration fluid was adjusted to the MTX infusion rate, targeting a total rate of 3000 mL/m^2^/24 h (MTX + rehydration fluid), as per the protocol [15].

The calcium folinate infusion rate and dose (given until s-MTX levels reach ≤ 0.2 µmol/L [15]) was determined by patient s-MTX levels and calculated using either patient BSA (if s-MTX < 5.0 µmol/L) or BW (if s-MTX ≥ 5.0 µmol/L) (Table 1). Due to the risk of severe adverse reactions following hypercalcemia [19,55,56], the upper limit for calcium folinate dose in children is 20 mg/kg [15], while the upper limit for the infusion rate is 50 mg/m^2^/min [57]. Therefore, administration time was as following: bolus (2–5 min) when dosed ≤ 20 mg/kg and ≤50 mg/m^2^/min or short infusion (60 min) when dosed > 20 mg/kg and >50 mg/m^2^/min. The y-site mixing ratios between calcium folinate and HCO_3_^−^-containing rehydration fluid were assumed to be directly proportional to the infusion rate ratios in the simulated models [48,49,50] (Figure 1).

### 2.3. Theoretical Analysis

#### 2.3.1. Defining Patient Models

Three sets of pediatric patient models (patient model 1, 2, and 3) were initially simulated using the y-site concentrations prediction model (Figure 1). The simulated patient models represented a set of pediatric patients, specifically children of approx. 1, 9, and 14 years of age, receiving rescue therapy on the second day of the protocol (Table 2).

In addition, a mix of 20 mL undiluted calcium folinate 10 mg/mL and 20 mL sodium bicarbonate (NaHCO_3_) 500 mmol/L (1:1 volume mix), referred to as undiluted mix, was tested for comparison with the simulated patient models. When calcium folinate dosing was used to estimate the age, BSA, and BW values in the case of the undiluted mix, this translated into values equivalent to an 11-year-old patient with a BSA of 1.3 m^2^, and BW of 40 kg (Table 2). Lastly, a hypothetical high-risk outlier control case, referred to as “extreme patient”, was included in the theoretical assessments to reference changes of effects in larger and older patients. Since children can vary tremendously in attributes (BW, BSA, etc.) both within the population but also within a certain age group, the extreme patient was added to assess how more extreme attributes would affect the protocol and the precipitation risk. In practice, the parameters corresponded to a 39-year-old patient. In the unlikely event that a pediatric patient with similar attributes were to be treated, this study also captures the outlier case.

Since calcium folinate and sodium bicarbonate are dosed based on BW and BSA, the estimation of these two parameters was necessary for compatibility simulations. In all cases, model BW was assigned based on expected weight for age, using growth charts that correlate age and BW in children [58,59]. Subsequently, BSA was assigned using charts deriving from the Du Bois formula [60], which describes a nonlinear relationship between BW, patient height, and BSA, where an increase in BW and height result in a relatively smaller increase in BSA.

For reference, the Aujoulat et al. [46] study tested the compatibility by mixing 2 mL of bicarbonate 167 mmol/L with 2 mL of calcium folinate 10 mg/mL. This corresponds to a predicted concentration of 8.35 × 10^−5^ mol/mL HCO_3_^−^ and 9.78 × 10^−6^ mol/mL Ca^2+^ in their final mix, and a 8.54 molar mixing ratio ([HCO_3_^−^]/[Ca^2+^]).

#### 2.3.2. Predicting Y-Site pH and CaCO_3_ Precipitation Risk

To evaluate the risk for CaCO_3_ precipitation in pediatric ALL rescue therapy, the maximum molar amounts and concentrations of HCO_3_^−^ and Ca^2+^ ions were calculated based on predicted mixing ratios (Table 2) in simulated patient model 1–3, the undiluted mix, and the “extreme patient” case, according to the y-site concentration prediction model (Figure 1). The final pH of the mixture was then estimated based on predicted HCO_3_^−^ concentration and its diprotic buffer system [61,62] (Supplementary Materials Figure S1) using the Henderson–Hasselbalch equation (Supplementary Materials Table S1). In these calculations, the conjugate acid concentration of carbonic acid (H_2_CO_3_, pKa 6.3 [63]) in the 40 mmol/L clinical NaHCO_3_-containing rehydration fluid solution was set to 0.11 mmol/L, which also corresponds to near-physiological pH (7.4). The resulting predicted free CO_3_^2−^ (pKa 10.3 [62,64]) and Ca^2+^ concentrations in the models and cases could then be compared with CaCO_3_ solubility (0.0166 mg/mL or 1.66 × 10^−7^ mol/mL at 20 °C [30]) in addition to their ionic products ([Ca^2+^] × [CO_3_^2−^], K_SP_ 3.36 × 10^−9^ mol^2^/L^2^ at 25 °C [65]), to evaluate whether precipitation could occur at the mixing concentrations expected at the y-site under clinical conditions. In addition to the protonation of carbonate and the solubility product, other ion-interaction effects that could have an influence on precipitation of CaCO_3_ were not considered in these calculations.

#### 2.3.3. Investigation of Model Representation of Clinical Scenarios

The concentrations used when identifying mixing ratios for the simulated patient models 1–3 were based on fixed s-MTX values (Table 2). In reality, patient s-MTX levels vary throughout the course of treatment, leading to dynamic changes in calcium folinate and HCO_3_^−^ [15,16,20]. In turn, this leads to variations in effective y-site mixing ratios, and thus intra-model variability regarding the potential to form CaCO_3_ precipitate.

In an attempt to evaluate what scenarios patient model 1–3 did account for, a series of simulations with variable s-MTX levels (constant BW and BSA maintained) were performed. The “extreme patient” outlier case was included to further reference changes of effects in patients with larger BSA and BW. The s-MTX levels were set to increase from 0.1 to 9.7 µmol/L in incremental steps of 0.1 µmol/L, in 97 steps. Correlated to this s-MTX increase, Ca^2+^ and HCO_3_^−^ concentrations were predicted based on protocol conditions and defined limits (Table 1 and Figure 1, respectively). Accordingly, Ca^2+^ infusion rates were configured to two minutes for patient model 1, 2, and the “extreme patient” case at s-MTX values ≤ 4.9 µmol/mL, and five minutes for patient model 3 at s-MTX values ≤ 7.5 µmol/mL. Above these s-MTX levels, model Ca^2+^ administration times were configured to 60 min for all models. pH changes were predicted based on variable s-MTX values. pH values ≥ 8.3 (i.e., midpoint between pK_a1_ 6.3 (H_2_CO_3_) and pK_a2_ 10.3 (HCO_3_^−^)), where the equilibrium shift was from HCO_3_^−^ towards CO_3_^2−^, was considered to be the determinant threshold value for CO_3_^2−^(aq) availability, potentially leading to an expected substantial increase in CaCO_3_(s) formation (Supplementary Materials Figure S1). Risk of precipitation at the y-site under these simulated conditions was then explored as described in Section 2.3.2.

### 2.4. Physical Compatibility Testing

Physical compatibility was tested in vitro and assessed for mixed samples corresponding to mixing ratios of the simulated patient models 1–3 (Table 2). In addition, an undiluted 1:1 volume mix of calcium folinate and sodium hydrogen carbonate was tested.

#### 2.4.1. Preparation of Samples

**Figure 2:**
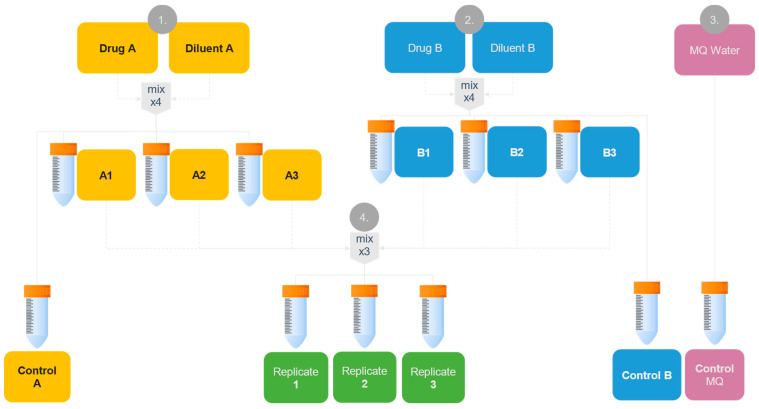
General compatibility testing program used for patient model 1–3, as recommended in the ALL-together treatment protocol [15]. Predicted volumes of three equal sets of A (A1, A2, and A3) and B (B1, B2, and B3) were added into three replicate tubes, representing the y-site mixing scenario for each patient. Diluent A was sodium hydrochloride 9 mg/mL and diluent B was a standard glucose–sodium–potassium infusion solution.

Calcium folinate 10 mg/mL in volumes of 0.73, 6.0, and 35.0 mL for simulated patient models 1–3, respectively, was diluted with sodium chloride 9 mg/mL to 40 mL in centrifuge tubes (A) (*n* = 4). In another tube (B), rehydration fluid was prepared by adding 3.2 mL sodium hydrogen carbonate 500 mmol/L to a standard glucose–sodium–potassium infusion solution to yield 40 mL of a rehydration fluid containing 40 mmol/L bicarbonate (*n* = 4). Varying proportions of A (38.8, 36.5, and 29.8 mL for patient models 1–3, respectively) to B (1.2, 3.5, and 10.2 mL for patient models 1–3, respectively) were mixed according to the mixing ratios identified in Table 2 (*n* = 3), simulating the y-site mixing scenario for each patient. Figure 2 illustrates the general flowchart for the testing program.

The undiluted mix of 1:1 volume ratio was prepared by adding 20 mL of calcium folinate 10 mg/mL and 20 mL sodium bicarbonate 500 mmol/L to a 40 mL centrifuge tube (*n* = 4). Additionally, 40 mL of unmixed A, and B, as well as MQ water served as controls throughout the analyses.

Samples were prepared in a particle-free environment inside a EuroFlow EF/S-4 safety cabinet (Clean Air^®^, Woerden, The Netherlands) at room temperature, according to antiseptic hospital workflow procedures [66,67]. All drug containers were carefully swirled 10 times before being used in preparations. Sterile 50 mL polypropylene (PP) centrifuge tubes with CentriStar™ PP caps (Corning™ Science, Tamaulipas, Mexico) were used as containers for the compatibility testing samples. Solutions were added to the tubes through a sterile 0.22 µm pore size syringe filter with a flat PES filter membrane (VWR International, Radnor Township, PA, USA) and effective filtration area of 2.8 cm^2^.

#### 2.4.2. Analysis of Physical Compatibility Using Validated Methods

The testing program to assess physical compatibility of the prepared samples (Figure 2) was performed immediately after mixing (t_0_) and 4 h after mixing (t_4_) according to previously established methods [51]. This included measurement of pH and assessment of potential particles through visual examination using a Tyndall beam, turbidity measurements, and sub-visual particle content using light obscuration. Additionally, the Tyndall effect was explored on samples 24 h after mixing (t_24_). All sample containers were carefully swirled 10 times just prior to analysis.

The risk of CaCO_3_(s) formation and thus precipitation increases at higher pH, particularly above pH 8 (see Supplementary Materials Figure S1). Hence, the pH value of the mixed samples provides an indication of the risk of particle formation. pH was measured using an InLab^®^ Versatile Pro electrode with a SevenCompact S220 pH-meter (Mettler-Toledo GmBH, Greifensee, Switzerland). Ionic products based on measured pH values were calculated for comparison with the calculated ionic products based on predicted pH (described in Section 2.3.2).

Tyndall beam was used to visually examine the presence of macroscopic particles in the samples, as explained earlier [49,51,68]. Mixed samples and controls were visually examined against a black background using a fiber-optic led beam (Schott KL 1600 LED, Mainz, Germany). Presence of Tyndall effect (visible coherent line through the sample) indicated particles, which may be attributed to CaCO_3_(s) precipitation if detected only in the mixed samples.

Sample turbidity was measured with a 2100Qis turbidimeter (Hach Lang, GmbH, Duesseldorf, Germany). An increase in Formazine Nephelometry Units (FNU) larger than 0.3 between controls and the mixed samples served as indication of particle formation in the mixed samples [51].

The sub-visual particle content was analyzed by light obscuration with the Accusizer Syringe Injection Sampler (PSS NICOMP, Billerica, MA, USA) to estimate particle size and amount. The samples were measured for three pulls of 5 mL of solution each, and the amounts of particles ≥ 5, 10 and 25 μm per mL were determined. Background count was defined as not more than 100 particles/mL ≥ 0.5 μm. As acceptance criteria, the limits for particle content in large-volume parenterals was used as described in the European Pharmacopoeia (Ph. Eur) Section 2.9.19; not more than 25 particles per mL of size ≥ 10 µm and not more than 3 particles per mL of size ≥ 25 µm. The ≥5 µm size measurement criterium was included based on the potential clinical relevance due to assumed capillary diameter size [49,51,68].

Since sub-visual particle content measurements were destructive, the testing program could not be performed on the same sample volumes. The two timepoints (t_0_ and t_4_) were therefore performed on two individual batches made in parallel. Tests on mixed samples were always compared to unmixed controls and MQ water.

### 2.5. Proofing Precipitate Identity Using Raman Microscopy

An alpha300 apyron confocal Raman microscopy system (WITec Wissenschaftliche Instrumente und Technologie GmbH, Ulm, Germany) equipped with a 532 nm laser source was used for microscopic and spectroscopic analysis of particle identity. Data acquisition and preprocessing was performed using the accompanying proprietary software bundle Suite SIX from WITec. The software TrueMatch v. 6.1.10.135 (WITec, Ulm, Germany) was used to compare acquired data to reference spectra.

#### 2.5.1. Preparation of Samples for Raman Analysis

Potential precipitates were collected from the samples for analysis in the Raman microscopy system using flat filter membranes at room temperature. It has been described in the literature that CaCO_3_ particles have a light color with a yellowish tint [19,40,42,46] and filter membrane color was assumed to affect the background contrast and thus the visibility of particles. Therefore, three different filters of different colors (white or black) were initially tested as follows: Supor^®^ PES (white), 0.2 µm pore size and 47 mm diameter (Pall Corporation, Ann Arbor, MI, USA) and Cyclopore™ PCTE black, 0.2 µm pore size and 25 mm diameter (Whatman™ Inc./Cytiva, Clifton, NJ, USA). The membranes were used for filtration with a 100 millibar VP100 vacuum pump (Vacuubrand GmBH, Wertheim, Germany) assembly system. Mixed samples with the highest and second highest predicted amounts of CaCO_3_ (undiluted mix and simulated Patient model 3), were chosen for analysis. The samples (5–10 mL) were filtered using different filter types: mixed samples from simulated patient model 3 were filtered with white PES and black PCTE membranes, A- and B-controls (Figure 2) were filtered with PCTE black membranes, and undiluted mix samples were filtered with PES membranes. Each filter was flushed through with 20–50 mL MQ water after sample filtration to remove unprecipitated material and then dried prior to Raman analysis.

Different reference materials were used to identify captured particles. Calcium carbonate standard was used to acquire a reference Raman spectrum for comparison with any precipitates collected from samples. In addition, a mixed sample corresponding to simulated patient model 3 was alkalized by addition of sodium hydroxide to pH >12 to force precipitation of CaCO_3_(s). This precipitate was then collected, analyzed, and used as a reference (denoted “patient model 3 alkalized”).

Reference spectra of calcium folinate and sodium hydrogen carbonate drug solutions were collected by deposition on microscope cover glass, measuring 24 × 50 × (0.13–0.17) mm (height × length × depth), (VWR International, Radnor Township, PA, USA), and allowed to air-dry prior to analysis. From these references, molecule specific high-resolution Raman spectra “fingerprints” were acquired and implemented into the local Raman spectra reference database for Hospital Pharmacies Enterprise at the Oslo University Hospital.

#### 2.5.2. Characterizing Particle Fingerprint for Proof of Identity

**Figure 3:**
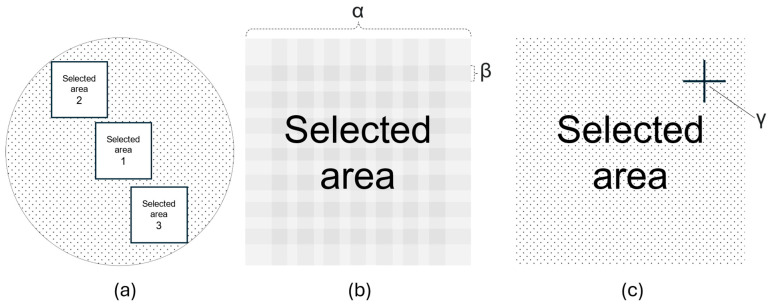
Schematic top-down view illustrations of Raman microscopical and spectroscopical investigation of filter membrane analysis. (**a**) Depiction of a porous filter membrane where three selected areas (1, 2, and 3) were video-stitched by 169 images, giving three larger images. One of these images is illustrated by (**b**), having a 1012 µm wide side (α) with a 40% overlap (β). (**c**) The larger video-stitched images were then examined and targeted for Raman spectrum analysis upon suspected particle discovery at this random point (γ).

The filter membranes were first inspected using photography with the Raman microscope equipped with a 20× magnification objective (Zeiss LD Plan-Neofluar 20×/0.4) over three randomly selected areas comprising 169 stitching images (40% cross-over border) spanning 1012 × 1012 µm as illustrated in Figure 3. Particle discovery on collected images was conducted by inspecting the stitched images manually.

Raman spectra were captured at any instance of suspected particle discovery within the selected areas with a 532 nm excitation wavelength laser at 2.5 mW power using a 600 g/mm BLZ 500.00 nm grating, integration time of 0.5 s, and 50 accumulations. Raman shifts in the spectral range from 0 to 4000 cm^−1^ were collected. Data preprocessing was conducted using the default state software settings, which includes cosmic ray removal and background subtraction. The recorded Raman spectra were compared to reference spectra across all selected areas per sample using the software TrueMatch (WITec, Ulm, Germany). The TrueMatch software evaluated their match mathematically and assigned a hit quality index (HQI) score accordingly. Spectra demonstrating a HQI score < 50 towards reference spectra, and spectra matching with respective filter membrane material (PES, PCTE), were defined as negative analysis results with respect to the presence of CaCO_3_ particles.

Any samples with HQI > 50 for any of the CaCO_3_ reference spectra in the commercial databases (S.T. JAPAN Europe GmBH, Cologne, Germany) were recorded with a 100× objective (Zeiss LD Plan-Neofluar 100×/0.9) and further compared to the CaCO_3_ standard, in-house reference spectra obtained for calcium folinate, sodium bicarbonate, and “patient model 3 alkalized”.

## 3. Results

### 3.1. Theoretical Analysis

#### 3.1.1. Predicted Ionic Yields, Molar Ratios and Risk of CaCO_3_ Precipitation

The predicted concentrations of calcium (Ca^2+^), bicarbonate (HCO_3_^−^), carbonate (CO_3_^2−^), and pH levels in the mixed samples, and the risk of CaCO_3_(s) precipitation for the simulated patient models based on their clinical parameters, dose, and infusion characteristics (Table 2) according to treatment conditions (Figure 1), are presented in Table 3. Looking at the molar HCO_3_^2−^/Ca^2+^ mixing ratio in the simulated patient models dictated by the protocol, a slight decrease from patient model 1 (3.56) to patient model 2 (1.32) and further to patient model 3 (0.80) was detected, i.e., decreasing with increasing age and size of the patient (Table 3). An increase in molar ratio was identified for the extreme patient model (5.82) as compared to patient model 3 (oldest and largest of the pediatric models). The undiluted mix (1:1 volume mix) was found to have a molar ratio that was almost 4× higher than patient model 1 at 12.8. The molar ratio found for the “extreme patient” and the undiluted mix would be more in the comparable range as reported in the study by Aujoulat et al., where their 1:1 mixing ratio of applied undiluted drugs [46] corresponded to a molar ratio of HCO_3_^2−^/Ca^2+^ of 8.54.

The final pH of the mixed samples was predicted to be near physiological (pH 7.4) for patient model 1, while reaching increasingly more alkaline values across the other models with higher HCO_3_^−^ content (Table 3). The predicted maximum theoretical CaCO_3_ concentration (the concentration of the least concentrated ionic component of the salt), based on estimated CO_3_^2−^ concentrations from predicted pH, was lower than the solubility of CaCO_3_ (1.66 × 10^−7^ mol/mL or 0.0166 mg/mL [30]) in patient models 1–3, but higher for the undiluted mix and “extreme patient” cases. Predicted ionic products were higher than the solubility product of CaCO_3_ (3.36 × 10^−9^ mol^2^/L^2^ [65]) in all but patient model 1 (Supplementary Materials Table S1). In total, only patient model 1 was conclusively assessed to avoid precipitation of CaCO_3_(s) based on both theoretical evaluations.

#### 3.1.2. Investigation of Model Representation of Clinical Scenarios

**Figure 4:**
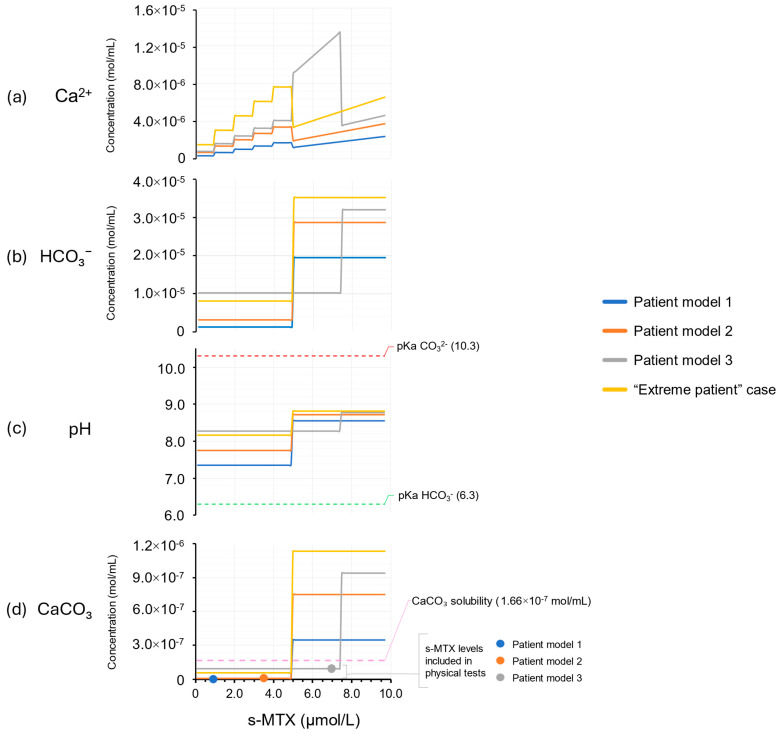
Visual model of equilibrium conditions and predictive check of concentrations in simulated y-site mixes of physically tested patient models 1–3 compared to the hypothetical “extreme patient” case, for (**a**) Ca^2+^, (**b**) HCO_3_^−^, (**c**) pH, and (**d**) CaCO_3_, when s-MTX increases (0.1–9.7 µmol/L). pH predictions were based on per-model predicted HCO_3_^−^ concentrations, when [H_2_CO_3_] is set to 0.11 mmol/L and CaCO_3_ concentrations were predicted based on estimated Ca^2+^ or CO_3_^2−^ concentrations, depending on which was the least available component. Dotted s-MTX values (4d) mark levels included in physical tests (Table 2).

The predictions presented in Table 3, and the identified mixing ratios, were based on fixed drug concentrations deriving from fixed s-MTX values assigned to the models (Table 2). However, this represents only a snapshot of the infusion regime according to the ALL-together protocol. To challenge this method and check whether the results represent all dynamically changing concentrations and mixing ratios encountered in the ALL-together protocol, stepwise increments of s-MTX for all simulated models and the “extreme patient” case were performed. In Figure 4, the predicted fluctuations in calcium folinate, HCO_3_^−^, CO_3_^2−^/Ca^2+^ molar ratio, and maximum CaCO_3_ yield (based on complete deprotonation of HCO_3_^−^ to CO_3_^2−^) in response to increasing s-MTX are illustrated for simulated patient models 1–3 and the “extreme patient” case. An incremental increase in Ca^2+^ concentration is expected (Figure 4a) with an abrupt shift at s-MTX 5 µmol/L and 7.5 µmol/L, respectively, as the ALL-together protocol [15] directs changes in calcium folinate administration time (bolus 2–5 min vs. short infusion 60 min) and infusion rates. When s-MTX concentrations rise above 5 µmol/L for patient model 1, 2, and “extreme patient”, or 7.5 µmol/L for patient model 3, a large increase in HCO_3_^−^ (Figure 4b) and pH is observed. Above these concentrations, predicted pH increases to above 8.3 for all patient models except patient model 3 (Figure 4c), where this shift occurs at s-MTX 7.5 µmol/L. At pH above 8.3, the fraction of dissolved carbonate in its monoprotonated form (HCO_3_^−^) decreases as it is converted into the fully deprotonated form (CO_3_^2−^) according to its pKa-values (6.3 and 10.3) and, therefore, there will be a significantly higher amount of dissolved CO_3_^2−^(aq) available, which in turn will increase the potential for precipitation of CaCO_3_(s). The increased pH in the mixes (Figure 4c) therefore corresponds to an increased risk of CaCO_3_(s) precipitation as the concentrations of CO_3_^−2^ reach levels above the solubility limit (Figure 4d). The mixing ratios identified based on fixed concentrations for patient model 1–3 (Table 2), further used in the physical compatibility tests, can all be found at maximum theoretical CaCO_3_ concentrations in solution below the solubility limit (Figure 4d) and should therefore be compatible.

### 3.2. Physical Compatibility Testing

#### 3.2.1. pH Measurements and Theoretical Consideration

Measured pH values in all simulated patient y-site mixes and controls are summarized in Table 4. The pH value of the mixed samples was typically between the two unmixed controls and corresponded to the mixing ratio of the two drugs. There was relatively good agreement between measured and predicted pH for patient model 1 and 2 (Table 3 and Table 4), though the measured pH was lower than the predicted values for patient model 3 and the undiluted mix.

The calculated ionic product values were consistently lower when using the measured pH (Table 4) compared to using the predicted pH (Table 3) though this did not change the final CaCO_3_(s) precipitation risk assessment. Only patient model 1 was found to have no risk of precipitation when comparing the calculated ionic products based on measured pH to the solubility product of CaCO_3_ (3.36 × 10^−9^ mol^2^/L^2^ [65]) (Supplementary Materials Table S1).

#### 3.2.2. Visual Examination Using Tyndall Light

Presence of macroscopic particles was visually examined using a Tyndall beam, and any presence of visible particles was documented (Table 5). All mixes based on simulated patient model concentrations were found to be particle-free, while the undiluted mix demonstrated distinctly visible particles within the first minutes upon mixing component A (calcium folinate solution) with B (sodium bicarbonate-containing rehydration fluid). An example of the precipitated particles (Figure S3 in Supplementary Material) is shown in the image of observed particles that stem from the undiluted mix, captured by the microscopy modality of the Raman microscope (100× objective). The estimated size of the particles was in the range 10–40 µm. A few dust particles and some bubbles were observed in some of the unmixed controls, but nothing indicating that the particles in the mixed samples originated from contamination of the solutions.

#### 3.2.3. Turbidimetry

Turbidity results are presented in Table 6. The turbidity of the mixed samples from simulated patient models resembled the turbidity of the unmixed controls, while the undiluted mixed sample demonstrated a considerably higher turbidity than its controls. An increase of more than 0.3 FNU in the mixed sample as compared to the unmixed controls was regarded as an indication of particle formation.

#### 3.2.4. Particle Size Measurement and Quantification

From the particle content measurements (size and amounts), all mixed samples simulating patient models 1–3, and their corresponding controls (A and B), were found to be within the Ph. Eur. criteria for large-volume parenterals of maximum 25 particles/mL ≥ 10 µm and not more than 3 particles/mL ≥ 25 µm (Table 7). Also, the number of particles in the size range corresponding to the small capillaries (≥5 µm) was low for these mixed samples. However, the undiluted mix samples showed a high number of particles of all sizes at t_0_ and the particle count could not be measured at t_4_ due to detector overload. These samples were not diluted to meet the detectable range of the instrument since a dilution step could dissolve particles.

#### 3.2.5. Characterizing Particle Fingerprint for Proof of Identity

Raman spectra of all materials were acquired and included in the local at-hospital reference database. This included reference spectra for the different filter membrane materials investigated to collect particles (PCTE and PES filter membranes), spectra for dried calcium folinate, sodium bicarbonate, powdered calcium carbonate standard, filtered sample of “patient model 3 alkalized” (i.e., forced CaCO_3_ precipitation) and undiluted mix samples.

From microscopy, the collected stitched images of filter membranes used to filter mixed samples corresponding to simulated patient model 3 using both PES (white) and black PCTE filter membranes exhibited particle-resembling artifacts in the images (Supplementary Materials Figure S2). These artifacts were identified as filter paper material (PES and PCTE) through analysis of the Raman spectra and the membranes did not show any visual indication of particles, suggesting that precipitation did not occur in these patient model experiments. In contrast, the PES filter used to filter the undiluted mix showed clear presence of particles on visual inspection. These particles were confirmed using Raman spectroscopy. It was found that the white background of the PES filters did not impede microscopical detection of particles. Though the undiluted mix was not tested on black PCTE filter membranes, other experiments showed that compared to the white PES filters, the black PCTE filter membranes provided a greater background contrast to artifacts. This could signify a possible advantage of the black PCTE filters over the PES filters when searching for particles.

**Figure 5:**
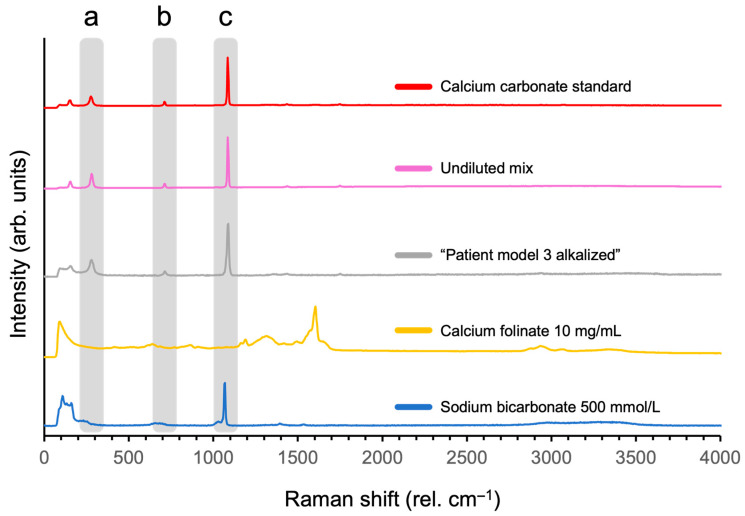
Raman spectra of the calcium carbonate standard, undiluted mix, “patient model 3 alkalized”, calcium folinate 10 mg/mL, and sodium bicarbonate 500 mmol/L. Shaded bands (a, b, and c) highlight peaks at similar positions (rel. cm^−1^) across samples. *Y*-axis consists of an arbitrary scale.

Raman spectra of CaCO_3_ standard and the undiluted mix sample matched with both the commercial and the in-house acquired reference spectra (HQI > 50) for calcium carbonate (Figure 5), with peak band positions at approximately 278, 712, and 1085 rel. cm ^−1^. Also, an alkalized sample of “patient model 3” at pH > 12 showed the same peaks. The dried sample of sodium bicarbonate (500 mmol/mL) included as a reference demonstrated a peak at 1067 rel. cm^−1^, though this peak did not show matching spectral peaks observed in spectra of the CaCO_3_ containing references. The Raman specter of calcium folinate did not have these corresponding peaks; instead, it had a peak at 1603 rel. cm^−1^.

## 4. Discussion

Our study demonstrates that calcium folinate and sodium bicarbonate are physiochemically compatible when co-administered in simulated patient models corresponding to patients of approximately 1, 9, and 14 years of age (Table 2) according to the ALL-together protocol [15]. We found that an increase in patient age (BW and BSA) or s-MTX—which led to an increase in calcium folinate dose—do not necessarily result in higher Ca^2+^ concentrations at y-site. Instead, an increase in HCO_3_^−^ concentrations and pH levels, due to protocol-directed changes in infusion rates, occurs. This fact, depending on the patient s-MTX value (Figure 4), may lead to precipitation of CaCO_3_(s) (Table 7) as confirmed by Raman microscopy (Figure 5). Predictive modelling does suggest that there is a certain and clinically relevant s-MTX range that allows for safe co-administration of calcium folinate and bicarbonate-containing rehydration fluid in pediatric ALL patients. This is a new finding.

The pediatric ALL-together protocol prescribes a complex and dynamic dosing regimen of calcium folinate combined with bicarbonate-containing rehydration fluid (Figure 1), during rescue therapy. According to our theoretical analysis, the HCO_3_^−^ content was in surplus in relation to Ca^2+^ in most models; Ca^2+^ was in surplus in relation to the CO_3_^2−^ content in all models except the undiluted mix (Table 3), demonstrating that pH conditions at the y-site shifted the equilibrium towards HCO_3_^−^ and not CO_3_^2−^, in the diprotic H_2_CO_3_/HCO_3_^−^/CO_3_^2−^ system (Supplementary Materials Figure S1). Only patient model 3, simulating a 14-year-old, was predicted to have more Ca^2+^ in relation to its HCO_3_^−^ content at y-site. This model had a Ca^2+^ concentration more than twice the concentration of the “extreme patient” case, simulating a 39-year-old patient. We attribute this to the applied Ca^2+^ infusion rate limits governed by the treatment protocol (shift at s-MTX ≥ 5 µmol/L), allowing for calcium folinate bolus injection (2 min) in patient model 3, but requiring the “extreme patient” to receive an infusion of 60 min instead. This also highlights the incompatibility risk for patients being treated close to the infusion rate limit of the protocol, necessitating a shift from bolus to short infusion (Table 1). Patient models 1–3 were assessed to represent safe patient scenarios for co-administration based solely on CaCO_3_ solubility and the lowest concentration component, but models 2 and 3 were found to be unsafe when calculating their predicted ionic products ([Ca^2+^] × [CO_3_^2−^]). In total, our theoretical risk assessment proposed that precipitation would not occur in patient model 1, while being inconclusive for models 2 and 3, and highly probable in the undiluted mix and the “extreme patient” case. The discrepancy between the two approaches (solubility vs. ionic product) observed here may be explained by the ionic product relying on both Ca^2+^ and CO_3_^2−^ concentrations, whereas the solubility-based approach instead relies on the limiting ingredient (either Ca^2+^ or CO_3_^2−^) determining the maximum theoretical CaCO_3_ yield. Here, for all models except the undiluted mix, CO_3_^2−^ was the limiting ingredient and determined the estimated resulting theoretical CaCO_3_ concentrations in solution. Thus, the solubility-based risk assessments conducted in this study depend less on predicted Ca^2+^ concentrations than our assessments based on the ionic product. Additionally, as CO_3_^2−^ is both a pH-dependent ingredient and a buffering agent, the ionic product may be more reliant on accurate pH estimations. This suggests that accurate pH estimations (and CO_3_^2−^ concentration predictions) may be of great importance for precipitation risk assessments. As confirmed by our physical testing (Table 3), pH was overestimated for all patient models (Table 4), leading to overestimation of the CO_3_^2−^ concentrations present in the mixes, resulting in an apparent ionic product that faultily indicates a precipitation risk. The same was observed when calculating the ionic product based on measured pH (Table 3), further demonstrating the complexity involved in accurately predicting the final mix pH for compatibility assessments of systems containing acids and bases.

The overestimation of pH and precipitation risk is confirmed by our physical tests, which showed that patient models 1–3 were compatible; all results from visual observation, along with particle count, turbidity, and pH measurements met the acceptance criteria [51,69]. Only the undiluted mix sample demonstrated massive precipitation within minutes of mixing, in line with its higher HCO_3_^−^ concentration resulting in a more alkaline solution and a higher concentration of CO_3_^2−^. As Ca^2+^ was available in the solution, the increasing presence of CO_3_^2−^ was expected to result in CaCO_3_ formation and precipitation. The particle count in the undiluted mix could not be estimated due to detector overload, indicating massive precipitation.

The identity of the particles from the undiluted mix could be verified as CaCO_3_ using Raman spectroscopy (Figure 5), and no other salts were identified in the dried sample solutions. The 1085 rel. cm^−1^ peak is highly prevalent in CaCO_3_ samples [70], while the sodium bicarbonate sample demonstrated a close peak at 1067 rel. cm^−1^, possibly due to it also containing a comparable carbonate structure [71]. The shift in 18 rel. cm^−1^ may be explained by the absence of Ca^2+^ ions in that sample or the protonation of the carbonate, resulting in a difference in vibrational transitions and Raman scattering [72].

When patient model 3 was forced to precipitate in high alkaline pH (above 12), CaCO_3_ formation was confirmed (Figure 5). This suggests that the HCO_3_^−^ and Ca^2+^ concentrations in patient model 3 indeed suffice for CaCO_3_ to form and precipitate, as long as the pH of the mixture shifts the equilibrium from HCO_3_^−^ into CO_3_^2−^.

Even though the in vitro tests provided clinically relevant simulations resulting in mixes absent of precipitates, they were based on fixed s-MTX levels giving fixed calcium folinate and sodium bicarbonate concentrations. However, this was expected to provide a snapshot and not the entire possible mixing panorama. In fact, our investigation of the scenario representativeness (Figure 4) demonstrated that when s-MTX increased above 5 µmol/L, only patient model 3 was estimated to remain below equilibrium conditions without precipitate. All other patient models reached the CaCO_3_ precipitation threshold (1.66 × 10^−7^ mol/mL [30]) at s-MTX 5.0 µmol/L, and patient model 3 at 7.5 µmol/L and above. At these s-MTX levels, adjusting the calcium folinate administration time from bolus (2–5 min) to short infusion (60 min) becomes necessary in order to ensure a Ca^2+^ load below 50 mg/m^2^/day [57] (Figure 1). This protocol-based change in administration time is explained by the ≥5.0 s-MTX level being the cut-off for switching to calcium folinate dosing based on BW × s-MTX instead of BSA × table value (Table 1). The changes result in dips in Ca^2+^ infusion rates, leading to increases in HCO_3_^−^ infusion rates instead, demonstrating the importance of considering the impact of s-MTX on fluid dynamic and y-site mixing ratios. Below s-MTX 5.0 µmol/L, the investigated scenarios (Figure 4) show that patient model 3, and thus the physically tested configuration, covered the high-risk outlier control “extreme patient” case, which suggests that co-administration is safe in pediatric patients with s-MTX levels below 5.0 µmol/L. Since rescue therapy aims at lowering s-MTX over time, and levels < 5.0 µmol/L are prevalent in children [20,73,74], this could be valuable information in clinical practice.

Our findings contradict those of Aujoulat et al. [46], who observed precipitates when mixing calcium folinate 10 mg/mL with bicarbonate 167 mmol/L in a 1:1 volume mixing ratio (i.e., 2 mL of each). While attempting to simulate pediatric rescue therapy, they mixed both components undiluted, which resulted in a pH of 7.37, visually observing precipitation after 30 min. However, according to our y-site concentration prediction model (Figure 1) this mix results in effective Ca^2+^ concentrations that are 23% lower and HCO_3_^−^ concentrations that are eight-fold higher than in patient model 3. Further, their molar mixing ratio ([HCO_3_^−^]/[Ca^2+^]) was 8.54 compared to 0.8 in our patient model 3, emphasizing a larger availability of bicarbonate and carbonate ions in their study protocol. To put this into perspective, our estimations suggest that such a mix would reach a pH of 9.18, exceeding the ionic product of all our patient models (1–3) (6.20 × 10^−5^ mol^2^/L^2^) and yielding a maximum theoretical CaCO_3_ concentration of 9.78 × 10^−6^ mol/mL, which would be 103 times higher than our patient model 3 (9.48 × 10^−8^ mol/mL). Unexpectedly, the Aujoulat study [46] also observed precipitation when mixing calcium folinate with a rehydration fluid of similar content and pH as used in the present study, but without any added bicarbonate. This incompatibility was attributed to pH effects, without addressing whether it was CaCO_3_ or calcium folinate that precipitated in their study. In the present study, the observed Raman peak bands in precipitates from samples aligned well with the literature [71,75,76], and CaCO_3_ was successfully identified as the precipitating agent from the undiluted mix and the alkalized patient model 3 (i.e., forced CaCO_3_ precipitation), while no precipitates of calcium folinate or other molecules were found. Furthermore, the investigated precipitate CaCO_3_ in our study proved to be an apt particle for exploring the performance of Raman spectroscopy and microscopy, with its characteristic 1085 cm^−1^ band peak being prominent throughout the analyses. This is further supported by this band peak being recommended for determining spectral resolution by Ph. Eur. [77]. As per conventional methods [78,79,80,81,82,83,84,85], the experiments conducted by Aujoulat et al. [46] assessed one single maximal dosing scenario and mixing ratio, whereas our study may be the first to assess Ca^2+^ compatibility with HCO_3_^−^ over a range of relevant patient scenarios, offering new insights that may instead enable safe co-administration under some common clinical circumstances.

To the best of our knowledge, this is the first study to systematically investigate the combined impact of patient clinical parameters on precipitation risk, by conducting extensive theoretical and physical compatibility testing, in an IV compatibility study. Furthermore, we augmented the analysis line-up [51] with the inclusion of an at-hospital Raman microscopy system, which allowed us to disclose the identity of formed precipitates and develop our Raman reference database further. Our approach, which mimics physiological conditions, provides clinically relevant insights that may improve multi-drug compatibility and administration guidelines in pediatric ALL treatment.

These findings highlight the importance of thoroughly understanding the impact of complex and dynamic treatment protocols on drug chemistry and pH when assessing physiochemical compatibility for clinical application [47]. Therefore, we advise future compatibility research to implement test setups that cover broader scenario representativeness, while assessing underlying mechanisms and verifying the identity of any precipitates formed. Co-administration must be conducted with utmost caution; nevertheless, our results indicate that mixing may be safe under certain conditions. If the two fluids could be given through the same venous catheter lumen (internal channel), it would reduce the number of venous access points needed to avoid mixing the drugs [86,87,88,89,90,91], enabling children to spend more time outside the clinic [43,92]. If the studied therapy were to be co-administered, it should be conducted through in-line filters to further safeguard the patients [93,94,95,96]. Such in-line filters are mandatory in pediatric care [97,98] but remain to be fully utilized in practice [99,100]. Another option for ensuring safe co-administration of bicarbonate during rescue therapy would be to choose disodium folinate [40,43,55] instead of calcium folinate. This alternative product has been shown to deliver equivalent therapy efficacy [40,101,102] and should eliminate the Ca^2+^ complexation-related precipitation risk altogether, but is often unavailable in the clinic, partly due to its higher pricing [43,55].

In summary, our study highlights the need for applying clinically relevant methods for compatibility assessments of parenteral drug solutions, which may contribute to optimizing administration strategies and therapeutic outcomes in pediatric cancer treatment. The Aujoulat study [46] is one of the major sources referred to when consulting calcium folinate and bicarbonate [44,45] compatibility and it has strongly impacted their co-administration practice over the years [17,44,45]. Here, instead of focusing on the worst-case scenario for mixing the two fluids, we directed our focus on the possibilities rather than limitations.

## 5. Limitations

One limitation of this study is that our theoretical prediction model assumed that calcium folinate is given diluted with sodium chloride 9 mg/mL as a 40 mL bolus, which in practice may vary in volume or even be given undiluted [57].

Another limitation may be that H_2_CO_3_ concentration was assigned a likely concentration (0.11 mmol/L) which may deviate from the actual concentration. In addition, the H_2_CO_3_/HCO_3_^−^ buffer system was expected to be the only factors driving the final pH value, excluding the potential impact from excipients and other additives on final pH in our samples. This was conducted to simplify pH estimations and CO_3_^2−^ predictions, which may explain the obtained overestimations.

Our models to predict pH and the risk of CaCO_3_ precipitation do not take into account the fluid dynamics in the clinical setting and the effects that mixing rates would have on these parameters. Both in the case of theoretical evaluation as well as physical testing, a dynamic model would be an improvement and make the models even more clinically relevant. To account for dynamic changes in the mixing ratio, we compensated by testing different mixing ratios more frequently. We aimed at using a worst-case scenario (undiluted mix) and a possible outlier case (extreme patient) in addition to typical pediatric patients (patient models 1–3). Furthermore, we investigated the dynamic aspect of the protocol in simulations described in Section 3.2.1.

Lastly, we conducted our physical experiments in a controlled laboratory setting that does not fully replicate clinical environments where changes in final Ca^2+^, HCO_3_^−^, and CO_3_^2−^ concentrations occur gradually as infusion rates are continuously adjusted.

## 6. Conclusions

Results from the physical compatibility tests of mixing ratios representing children of approximately 1, 9, and 14 years of age showed that doses and infusion rates of calcium folinate and bicarbonate-containing rehydration fluids in the ALL-together protocol were compatible, and the two fluids can be safely co-administered via the same lumen (internal channel) in these simulated patient models. The application of Raman spectroscopy confirmed the identity of precipitates from a non-patient model case.

Theoretical tests challenging the experimental area showed that situations where the two fluids are not compatible also do exist within the protocol framework, and precipitation of CaCO_3_ must be expected under some clinically relevant conditions. Risks of precipitation were identified in situations where s-MTX was above 5 µmol/L and administration of calcium folinate had to be given as short infusion (1 h) instead of a bolus injection (2–5 min). In such situations, co-administration in the same lumen must be avoided.

These novel findings, correlating the clinical parameter s-MTX levels to resulting y-site concentrations, analyzed with Raman spectroscopy, advance compatibility research by offering precise chemical insights and predictive modeling capabilities to compatibility testing, thus facilitating relevant co-administration information to pediatric rescue therapy. The study’s limitations include assumptions regarding bolus volume variability, pH predictions, exclusion of excipient effects on pH estimations, and conducting experiments in controlled environments that may not fully replicate clinical conditions.

Future research should continue to explore the interplay between clinical parameters and precipitation risk, focusing on chemical interactions when tested drugs are mixed. Compatibility information should be based on diverse, clinically relevant scenarios to become applicable and add value in pediatric oncology protocols.

## Figures and Tables

**Table 1:** Calcium folinate dosing based on patient serum methotrexate concentration (s-MTX) from hour 48 and onwards, according to the pediatric ALL-together protocol [15]. Calcium folinate is administered until s-MTX ≤ 0.2 µmol/L, as a bolus (2–5 min) when dosed ≤ 20 mg/kg [15] and ≤50 mg/m^2^/min [57] or short infusion (60 min) when dosed > 20 mg/kg and >50 mg/m^2^/min.

s-MTX (µmol/L)	Calcium Folinate (mg/m^2^)
<1.0	15
1.0–1.9	30
2.0–2.9	45
3.0–3.9	60
4.0–4.9	75
≥5.0	s-MTX × BW *

* BW: Bodyweight.

**Table 2:** Assigned key clinical parameters (bodyweight, body surface area, and serum methotrexate concentration (s-MTX) of the simulated pediatric patient models 1–3 and undiluted mix, together with the predicted infusion rates of calcium folinate and HCO_3_^−^-containing rehydration fluid and their estimated mixing ratios. The “extreme patient” case was added to the rest of the study lineup as a potential high-risk outlier for reference and therefore does not correspond to a typical pediatric patient.

	Physical and Theoretical Testing				Theoretical Testing
	Patient Model 1	Patient Model 2	Patient Model 3	Undiluted Mix ^a^	Extreme Patient
Age (years)	1	9	14	11 ^d^	39
Bodyweight (kg)	10	28	50	40 ^d^	118
Body surface area (m^2^)	0.49	1.0	1.5	1.3 ^d^	2.6
s-MTX (µmol/L)	0.9	3.5	7.0	5.0 ^d^	9.0
Calcium folinate dose (mg)	7.35	60	350	200	1062
NaHCO_3_ dose (mmol)	0.050	0.14	0.41	10.0	1.41
Administration time (min)	2.0	2.0	5.0	60 ^d^	60
Calcium folinate infusion rate (mL/h)	1200	1200	480	1200 ^d^	40.0
Rehydration fluid ^b^ infusion rate (mL/h)	38.3	116	165	151 ^d^	302
Mixing ratio ^c^	31.3	10.3	2.92	1.00	0.13

^a^ 1:1 ratio of undiluted calcium folinate 10 mg/mL (20 mL) and NaHCO_3_ 500 mmol/L (20 mL); ^b^ containing NaHCO_3_; ^c^ calculated as infusion rate of calcium folinate over infusion rate of NaHCO_3_-containing rehydration fluid; ^d^ corresponding value when extrapolating the tested calcium folinate dose (Table 1) of undiluted mix model.

**Table 3:** Predicted content of Ca^2+^(aq) from calcium folinate, and HCO_3_^−^(aq) from the sodium bicarbonate-containing rehydration fluid., their relative molar ratio and pH values based on the simulated patient models 1–3, the undiluted mix, and the “extreme patient” case. Their theoretical risk for CaCO_3_(s) precipitation in mixed samples (40 mL) are based on solubility and ionic product (Supplementary Materials Table S1). Predictions are based on their assigned clinical parameters, dose, and infusion parameters (Table 2) and mixing protocol according to treatment conditions described in Figure 1.

	Patient Model 1	Patient Model 2	Patient Model 3	Undiluted Mix	Extreme Patient
[Calcium folinate](aq) (mg/mL)	0.18	1.37	6.52	5.00	3.10
[Ca^2+^](aq) (mol/mL)	3.48 × 10^−7^	2.67 × 10^−6^	1.27 × 10^−5^	1.96 × 10^−5^	6.07 × 10^−6^
[HCO_3_^−^](aq) (mol/mL)	1.24 × 10^−6^	3.52 × 10^−6^	1.02 × 10^−5^	2.50 × 10^−4^	3.53 × 10^−5^
Ca^2+^(aq) amount (mol)	1.39 × 10^−5^	1.07 × 10^−4^	5.10 × 10^−4^	7.82 × 10^−4^	2.43 × 10^−4^
HCO_3_^−^(aq) amount (mol)	4.95 × 10^−5^	1.41 × 10^−4^	4.09 × 10^−4^	1.00 × 10^−2^	1.41 × 10^−3^
HCO_3_^−^/Ca^2+^ molar ratio	3.56	1.32	0.80	12.8	5.82
Predicted pH	7.35	7.81	8.27	9.66	8.81
[CO_3_^2−^](aq) (mol/mL) *	1.39 × 10^−9^	1.13 × 10^−8^	9.48 × 10^−8^	5.68 × 10^−5^	1.13 × 10^−6^
CO_3_^2−^/Ca^2+^ molar ratio *	4.00 × 10^−3^	4.22 × 10^−3^	7.44 × 10^−3^	2.91	1.87 × 10^−1^
Maximum theoretical [CaCO_3_](aq or s) (mol/mL) **	1.39 × 10^−9^	1.13 × 10^−8^	9.48 × 10^−8^	1.96 × 10^−5^	1.13 × 10^−6^
CaCO_3_(s) precipitation risk based on solubility ***	no	no	no	yes	yes
Ionic product [Ca^2+^] × [CO_3_^2−^] (mol^2^/L^2^) *	4.85 × 10^−10^	3.02 × 10^−8^	1.21 × 10^−6^	1.11 × 10^−3^	6.88 × 10^−6^
CaCO_3_(s) precipitation risk based on ionic product ****	no	yes	yes	yes	yes
Total assessment of CaCO_3_(s) precipitation risk	no	inconclusive	inconclusive	yes	yes

* Based on predicted pH; ** Understood as the theoretical concentration of formable salt (CaCO_3_) in solution. This is therefore equal to the concentration of the least concentrated ionic component of the salt; *** Maximum theoretical [CaCO_3_](aq or s) compared to CaCO_3_ solubility (1.66 × 10^−7^ mol/mL or 0.0166 mg/mL [30]); **** Ionic product compared to solubility product (K_sp_) of CaCO_3_ (3.36 × 10^−9^ mol^2^/L^2^ [65]).

**Table 4:** The measured pH values (mean ± SD) of mixed solutions of simulated patient model 1–3 and the undiluted mix, in addition to their corresponding control samples A-control (calcium folinate) and B-control (sodium bicarbonate-containing rehydration fluid). Ionic products based on measured pH at t_0_ and t_4_ were included for comparison with the ionic products based on predicted pH in Table 3. Detailed calculations are presented in Supplementary Materials Table S1.

		y-Site Mix	A-Control	B-Control	Ionic Product * (mol^2^/L^2^)	CaCO_3_(s) Precipitation Risk **
		(*n* = 3)	(*n* = 1)	(*n* = 1)		
Patient model 1	t_0_	7.29 ± 0.02	6.44	8.36	4.21 × 10^−10^	No
	t_4_	7.30 ± 0.05	6.45	8.38	4.31 × 10^−10^	
	pH change	0.01	0.01	0.02		
Patient model 2	t_0_	7.63 ± 0.05	6.71	8.20	2.01 × 10^−8^	Yes
	t_4_	7.68 ± 0.06	6.69	8.27	2.26 × 10^−8^	
	pH change	0.05	0.02	0.07		
Patient model 3	t_0_	7.73 ± 0.01	7.07	8.20	3.50 × 10^−7^	Yes
	t_4_	7.74 ± 0.01	7.05	8.21	3.58 × 10^−7^	
	pH change	0.01	0.02	0.01		
Undiluted mix	t_0_	7.51 ± 0.03	7.34	8.18	7.93 × 10^−6^	Yes
	t_4_	7.98 ± 0.11	7.31	8.15	2.34 × 10^−5^	
	pH change	0.62	0.04	0.02		

* Ionic product of [Ca^2+^] × [CO_3_^2−^], based on measured pH; ** Ionic product compared to solubility product (K_sp_) of CaCO_3_ (3.36 × 10^−9^ mol^2^/L^2^ [65]).

**Table 5:** The results from visual examination of simulated y-site mixes using Tyndall beam of patient model 1–3 and the undiluted, in addition to their corresponding control samples, A-control (calcium folinate) and B-control (sodium bicarbonate-containing rehydration fluid), at timepoints t_0_, t_4_, and t_24_, respectively.

		y-Site Mix (*n* = 3)	A-Control (*n* = 1)	B-Control (*n* = 1)
	t_0_	clear	clear	clear
Patient model 1	t_4_	clear	clear	clear
	t_24_	clear	clear	clear
	t_0_	clear	clear	clear
Patient model 2	t_4_	clear	clear	clear
	t_24_	clear	clear (dust)	clear (dust)
	t_0_	clear (yellow)	clear (yellow)	clear
Patient model 3	t_4_	clear (yellow)	clear (yellow)	clear (bubbles)
	t_24_	clear (yellow)	clear (yellow)	clear (bubbles)
	t_0_	visible particles (yellow)	clear (yellow and bubbles)	clear (bubbles)
Undiluted mix	t_4_	visible particles (yellow)	clear (yellow)	clear
	t_24_	visible particles (yellow)	clear (yellow)	clear (dust)

**Table 6:** Turbidimetric evaluations showing formazine nephelometry units (FNU) mean ± SD values for simulated y-site mixes of patient models 1–3 and the undiluted mix, in addition to their corresponding control samples A-control (calcium folinate) and B-control (sodium bicarbonate-containing rehydration fluid), and MQ water (particle-free water) from timepoint t_0_ and t_4_.

		y-Site Mix (*n* = 3)	A-Control (*n* = 1)	B-Control (*n* = 1)	MQ Water (*n* = 1)
Patient model 1	t_0_	0.13 ± 0.02	0.12	0.14	0.11
	t_4_	0.14 ± 0.02	0.12	0.17	0.14
Patient model 2	t_0_	0.18 ± 0.10	0.29	0.16	0.19
	t_4_	0.16 ± 0.02	0.21	0.19	0.18
Patient model 3	t_0_	0.29 ± 0.04	0.48	0.28	0.27
	t_4_	0.18 ± 0.06	0.18	0.17	0.16
Undiluted mix	t_0_	62.4 ± 24.5	0.24	0.73	0.30
	t_4_	81.4 ± 96.0	0.32	0.87	0.14

**Table 7:** Particle concentration (#amount/mL) in fractions ≥ 5 µm, ≥10 µm, and ≥25 µm in simulated y-site mixes of patient model 1–3 and undiluted mix, in addition to their corresponding control samples, A-control (calcium folinate) and B-control (sodium bicarbonate-containing rehydration fluid, at timepoints t_0_ and t_4_ given as mean ± SD. Some samples could not be measured due to detector overload (OL).

		y-Site Mix (*n* = 9)		A-Control (*n* = 3)		B-Control (*n* = 3)	
		t_0_	t_4_	t_0_	t_4_	t_0_	t_4_
Patient model 1	≥5	0.22 ± 0.40	0.40 ± 0.41	0.13 ± 0.12	1.20 ± 0.87	0.33 ± 0.46	0.27 ± 0.46
	≥10	0.20 ± 0.40	0.27 ± 0.26	0.13 ± 0.12	0.80 ± 0.69	0.20 ± 0.20	0.27 ± 0.46
	≥25	0.09 ± 0.18	0.09 ± 0.15	0.07 ± 0.12	0.20 ± 0.20	0.00 ± 0.00	0.00 ± 0.00
Patient model 2	≥5	1.69 ± 1.29	5.71 ± 3.99	1.20 ± 0.53	6.33 ± 1.10	1.20 ± 1.06	0.67 ± 0.50
	≥10	0.22 ± 0.31	2.51 ± 1.98	0.27 ± 0.31	2.67 ± 1.01	0.13 ± 0.12	0.27 ± 0.12
	≥25	0.02 ± 0.07	0.38 ± 0.34	0.07 ± 0.12	0.20 ± 0.20	0.00 ± 0.00	0.00 ± 0.00
Patient model 3	≥5	1.58 ± 1.61	1.56 ± 1.06	0.53 ± 0.31	0.80 ± 0.20	0.40 ± 0.35	1.00 ± 0.87
	≥10	0.53 ± 0.46	0.69 ± 0.54	0.33 ± 0.31	0.20 ± 0.35	0.13 ± 0.35	0.47 ± 0.64
	≥25	0.11 ± 0.15	0.13 ± 0.17	0.13 ± 0.23	0.13 ± 0.23	0.00 ± 0.00	0.07 ± 0.12
Undiluted mix	≥5	4070 ± 2320	OL	2.27 ± 1.17	OL	6.20 ± 1.51	OL
	≥10	345 ± 160	OL	1.13 ± 1.01	OL	0.60 ± 0.20	OL
	≥25	24.9 ± 15.1	OL	0.07 ± 0.12	OL	0.07 ± 0.12	OL

## Data Availability

The original contributions presented in this study are included in the article/Supplementary Material. Further inquiries can be directed to the corresponding author(s). The raw data supporting the conclusions of this article will be made available by the authors on request.

## Supplementary Materials

The following supporting information can be downloaded at https://www.mdpi.com/article/10.3390/pharmaceutics17091155/s1; Figure S1: Visualization of the diprotic activity of carbonic acid in relation to pH in aqueous solution; Figure S2: Proofing particle identity with Raman spectroscopy; Figure S3: Discovered particles form the undiluted mix retained by a polyethersulfone filter membrane. The image was taken with the Raman microscope equipped with the 100× objective. Estimated diameter of the largest particle approximately 35–40 µm; Table S1: Predicted pH based on calculations using the Henderson–Hasselbalch equation and ionic product calculations based on predicted and measured pH, respectively.

## Abbreviations

The following abbreviations are used in this manuscript:

ALL	Acute lymphoblastic leukemia
IV	Intravenous(ly)
s-MTX	Serum methotrexate concentrations
CVC	Centrally inserted venous catheter
PICC	Peripherally inserted central venous catheter
MQ	Milli-Q^®^
PES	Polyethersulfone
BW	Bodyweight
BSA	Body surface area
PP	Polypropylene
FNU	Formazine Nephelometry Units
Ph. Eur.	European Pharmacopoeia
PCTE	Track-etched polycarbonate
HQI	Hit quality index
OL	Overload
