# Supplementary material for: Strategies for Assessing Physical Compatibility of Calcium Folinate with Bicarbonate During Methotrexate Rescue Therapy in Pediatric Patients with Acute Lymphoblastic Leukemia
Source: Pharmaceutics. 2025 Sep 3;17(9):1155. doi: 10.3390/pharmaceutics17091155 (PMC12472967; doi:10.3390/pharmaceutics17091155)
Supplement: Supplementary file 1 [file pharmaceutics-17-01155-s001.zip › pharmaceutics-3796589-supplementary.pdf]

## Supplementary Materials

# Strategies for assessing physical compatibility of calcium folinate with bicarbonate during methotrexate rescue therapy in pediatric patients with acute lymphoblastic leukemia

**Kaveh Teimori [1, 2, *], Bjarke Strøm Larsen [2], Mathias Buaas Austli [1, 3], Niklas Nilsson [1, 3], Ingunn Tho [2, *], and Katerina Nezvalova-Henriksen [1, 2, 3]**

[1] Oslo Hospital Pharmacy, Hospital Pharmacies Enterprise, South-Eastern Norway, Oslo 0372, Norway; kaveh.teimori@sahf.no (K.T), mathias.buaas.austli@sahf.no (M.B.A); niklas.nilsson@sahf.no (N.N), katerina.nezvalova-henriksen@sahf.no (K.N-H)

[2] Department of Pharmacy, University of Oslo, Oslo 0316, Norway; b.s.larsen@farmasi.uio.no (B.S.L), ingunn.tho@farmasi.uio.no (I.T)

[3] Oslo University Hospital, South-Eastern Norway, Oslo 0372, Norway

* Correspondence: kaveh.teimori@sahf.no; ingunn.tho@farmasi.uio.no

*Contents of supplementary information*

- Figure S1: Visualization of the diprotic activity of carbonic acid in relation to pH in aqueous solution.
- Figure S2: Proofing particle identity with Raman spectroscopy.
- Figure S3: Discovered particles form the undiluted mix retained by a polyethersulfone filter membrane. The image was taken with the Raman microscope equipped with the 100x objective. Estimated diameter of the largest particle approximately 35-40 μm.
- Table S1: Predicted pH based on calculations using the Henderson-Hasselbalch equation and ionic product calculations based on predicted and measured pH, respectively.

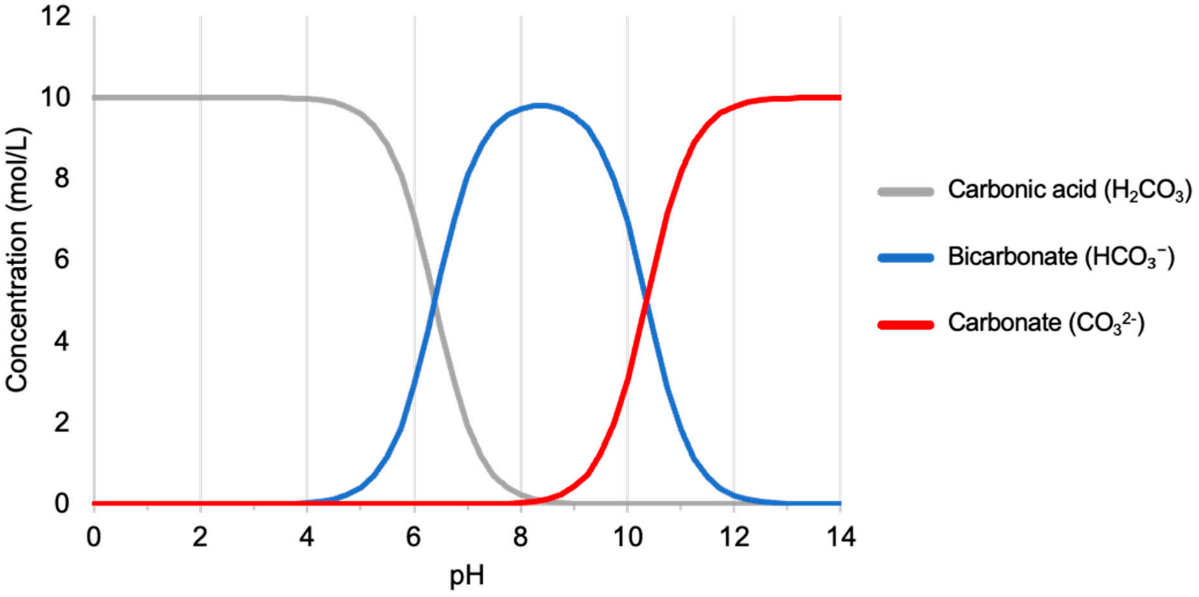


**Figure S1.** Visualization of the diprotic activity of carbonic acid (pKa 6.3, [1]) in relation to pH in aqueous solution. The reversible deprotonation of carbonic acid (H2CO3) into hydrogen carbonate ($HCO_3^-$) occurs theoretically above pH 4.3 and reaches 50% (exemplified here by 5 mol/L) when pH equals the pKa-value of $HCO_3^-$ (6.3). As pH reaches 8.3, H2CO3 is completely deprotonated and the $HCO_3^-$ concentration peaks (exemplified here by 10 mol/L). At pH 8.3 and above, the equilibrium shifts into further deprotonation of $HCO_3^-$ into carbonate ($CO_3^{2-}$), reaching a 50% mixture of both $HCO_3^-$ and $CO_3^{2-}$ at pH 10.3, the pKa of $CO_3^{2-}$. Complete theoretical deprotonation of $HCO_3^-$ occurs at pH 12.3.

Reference:
[1] NCBI. *Sodium Bicarbonate Compound Summary—Dissociation Constants*; NCBI: Bethesda, MD, USA, 2005

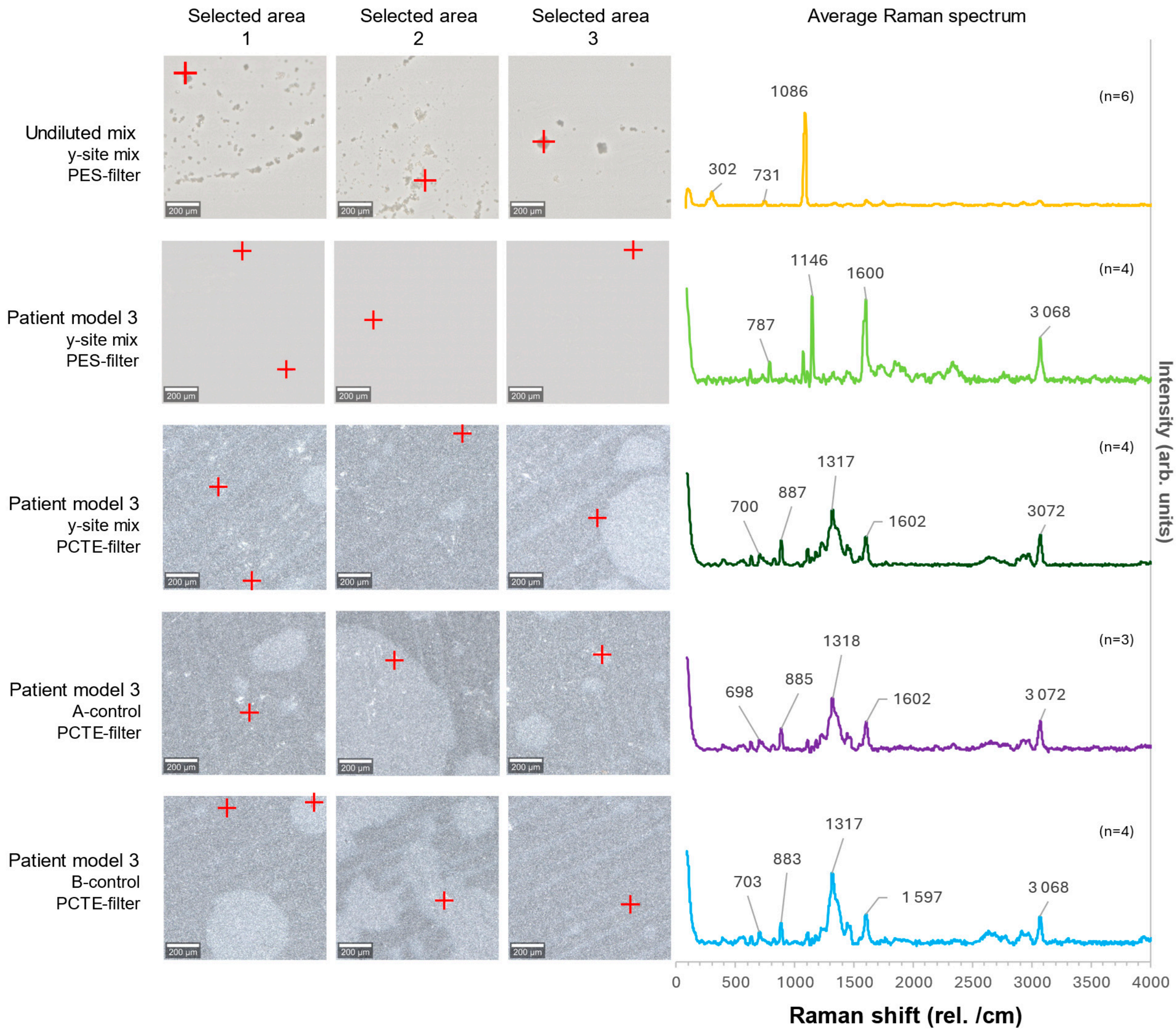


**Figure S2.** Proofing particle identity with Raman spectroscopy. Overview of images of selected areas 1-3, composed of 169 video-stitched images (1012x1012 μm) captured with the 20x objective. The plus-sign(s) on each image indicates the location of the spot targeted for Raman spectroscopy analysis. The far-right column summarizes averaged Raman spectra for the respective samples. The "undiluted mix" and patient model 3 on PES filter membrane demonstrate the visual difference between a positive vs a negative analysis result. Patient model 3 on black PCTE membrane demonstrates how artifacts arise randomly regardless of sample content differences, A-control appearing nearly visually identical. The Raman spectra of the "undiluted mix" sample scored > 81.9 on HQI for calcium carbonate from the "patient model 3 alkalized" sample. Raman spectra of the particles were averaged (n=6) to establish the spectroscopical profile as a positive fingerprint. Patient model 3 on both PES and PCTE, and its A- and B-controls were accordingly analyzed for Raman spectra, averaged and compared. It was found that suspected spots identified as "potential particles" were consistent with the filter materials based on hits with library reference spectrum: The patient model 3 on PES membrane had an average Raman spectra (n=4) hitting >90.6 HQI for PES, patient model 3 on black PCTE membrane hit >73.6 for a PCTE filter membrane, both A- and B-control samples at >58.5 and >70.9 averaging (n=3 and 4, respectively) a positive match for PCTE at their suspected spots.

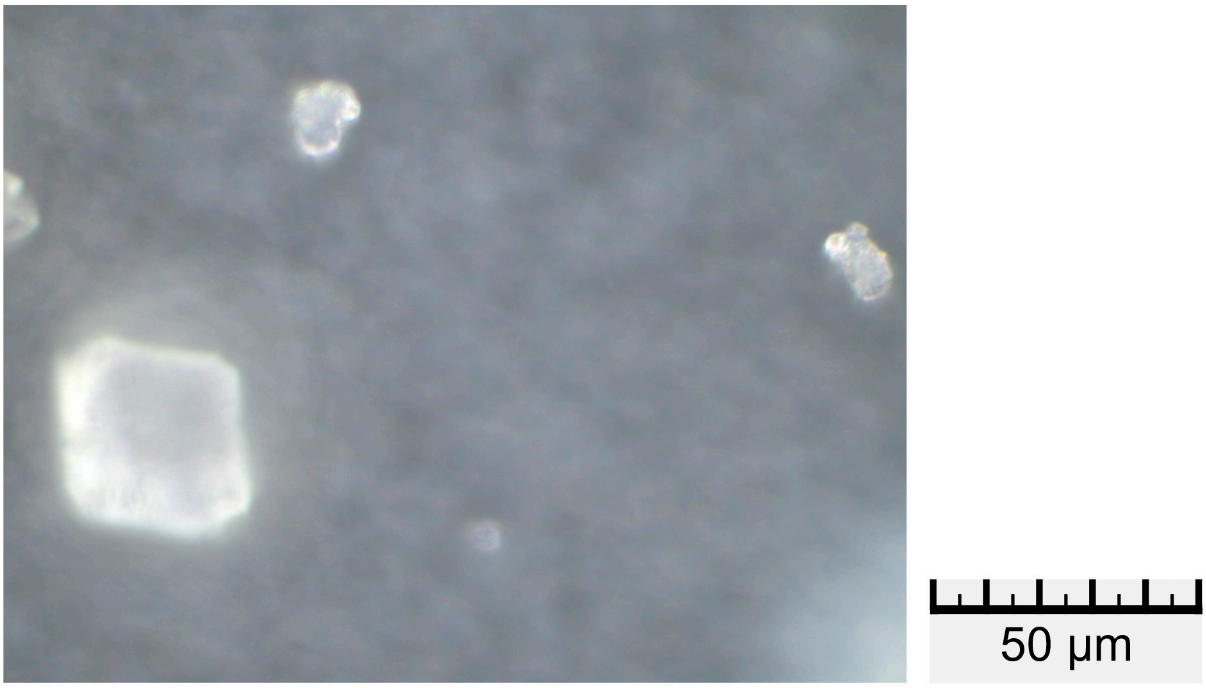


**Figure S3**: Discovered particles form the undiluted mix retained by a polyethersulfone filter membrane. The image was taken with the Raman microscope equipped with the 100x objective. Estimated diameter of the largest particle approximately 35-40 µm.

**Table S1.** Predicted pH based on calculations using the Henderson-Hasselbalch equation and ionic product calculations based on predicted and measured pH, respectively.

| | Patient model 1 | Patient model 2 | Patient model 3 | Undiluted mix | Extreme patient |
|---|---|---|---|---|---|
| *pH predictions based on estimated* $[HCO_3^-]$ | | | | | |
| Included $[H_2CO_3]$ value (mol/L) | $1.10 \times 10^{-4}$ | $1.10 \times 10^{-4}$ | $1.10 \times 10^{-4}$ | $1.10 \times 10^{-4}$ | $1.10 \times 10^{-4}$ |
| Total volume of mix (L) | 0.04 | 0.04 | 0.04 | 0.04 | 0.04 |
| Final $[HCO_3^-]$ in mix (mol/L) | $1.24 \times 10^{-3}$ | $3.52 \times 10^{-3}$ | $1.02 \times 10^{-2}$ | 0.25 | $3.53 \times 10^{-2}$ |
| pKa $HCO_3^-$ | 6.30 | 6.30 | 6.30 | 6.30 | 6.30 |
| pH predicted* | 7.35 | 7.81 | 8.27 | 9.66 | 8.81 |
| *Ionic product based on predicted pH* | | | | | |
| $[CO_3^{2-}]$ in mix (mol/L)** | $1.39 \times 10^{-6}$ | $1.13 \times 10^{-5}$ | $9.48 \times 10^{-5}$ | $5.68 \times 10^{-2}$ | $1.13 \times 10^{-3}$ |
| $[Ca^{2+}]$ in mix (mol/L) | $3.48 \times 10^{-4}$ | $2.67 \times 10^{-3}$ | $1.27 \times 10^{-2}$ | $1.96 \times 10^{-2}$ | $6.07 \times 10^{-3}$ |
| Ionic product ($mol^2/L^2$)*** | $4.85 \times 10^{-10}$ | $3.03 \times 10^{-8}$ | $1.21 \times 10^{-6}$ | $1.11 \times 10^{-3}$ | $6.88 \times 10^{-6}$ |
| *Ionic product based on measured pH at* $t_0$ | | | | | |
| pH measured $t_0$ | 7.29 | 7.63 | 7.73 | 7.51 | - |
| pKa $CO_3^{2-}$ | 10.30 | 10.30 | 10.30 | 10.30 | - |
| $[CO_3^{2-}]$ in mix (mol/L)** | $1.21 \times 10^{-6}$ | $7.53 \times 10^{-6}$ | $2.75 \times 10^{-5}$ | $4.05 \times 10^{-4}$ | - |
| $[Ca^{2+}]$ in mix (mol/L) | $3.48 \times 10^{-4}$ | $2.67 \times 10^{-3}$ | $1.27 \times 10^{-2}$ | $1.96 \times 10^{-2}$ | - |
| Ionic product ($mol^2/L^2$)*** | $4.21 \times 10^{-10}$ | $2.01 \times 10^{-8}$ | $3.50 \times 10^{-7}$ | $7.93 \times 10^{-6}$ | - |
| *Ionic product based on measured pH at t4* | | | | | |
| pH measured $t_4$ | 7.30 | 7.68 | 7.74 | 7.98 | - |
| pKa $CO_3^{2-}$ | 10.30 | 10.30 | 10.30 | 10.30 | - |
| $[CO_3^{2-}]$ in mix (mol/L)** | $1.24 \times 10^{-6}$ | $8.45 \times 10^{-6}$ | $2.81 \times 10^{-5}$ | $1.20 \times 10^{-3}$ | - |
| $[Ca^{2+}]$ in mix (mol/L) | $3.48 \times 10^{-4}$ | $2.67 \times 10^{-3}$ | $1.27 \times 10^{-2}$ | $1.96 \times 10^{-2}$ | - |
| Ionic product ($mol^2/L^2$)*** | $4.31 \times 10^{-10}$ | $2.26 \times 10^{-8}$ | $3.58 \times 10^{-7}$ | $2.34 \times 10^{-5}$ | - |

*pH = 6.3 + log ($[HCO_3^-]/[H_2CO_3]$) (the Henderson-Hasselbalch equation), **$[HCO_3^-] \times 10^{pH-10.3}$, ***$[Ca^{2+}] \times [CO_3^{2-}]$
